# Gluten Polymer Networks—A Microstructural Classification in Complex Systems

**DOI:** 10.3390/polym10060617

**Published:** 2018-06-05

**Authors:** Isabelle Lucas, Thomas Becker, Mario Jekle

**Affiliations:** Research Group Cereal Technology and Process Engineering, Institute of Brewing and Beverage Technology, Technical University of Munich, 85354 Freising, Germany; isabelle.lucas@tum.de (I.L.); tb@tum.de (T.B.)

**Keywords:** CLSM, protein network analysis, microstructure, wheat, gluten, network type

## Abstract

A classification of gluten polymer networks would support a better understanding of structure-function relationships of any gluten polymer material and thus, the control of processing properties. However, quantification and interpretation of the gluten network structures is challenging due to their complexity. Thus, the network formation was altered by specific gluten-modifying agents (glutathione, ascorbic acid, potassium bromate, glucose oxidase, transglutaminase, bromelain) in this study in order to clarify if structural alterations can be detected on a microstructural level and to specify different polymer arrangements in general. Microstructure analysis was performed by confocal laser scanning microscopy followed by quantification with protein network analysis. It was shown that alterations in gluten microstructure could be elucidated according to the kind of modification in cross-linking (disulphide, (iso) peptide, dityrosyl). Linear correlations of structural network attributes among each other were found, leading to an assertion in general: the higher the branching rate, the thinner the protein threads and the larger the interconnected protein aggregate. Considering the morphological attribute lacunarity, a quantitative classification of different gluten arrangements was established. These assertions were extended by using unspecific gluten-modifying agents in addition to the specific ones. Ultimately, five network types were proposed based on diverse polymer arrangements.

## 1. Introduction

Wheat gluten polymer does not only play an important role as a main structural component in wheat dough or bread, it is also applied in biomaterials like films, gels, foams, or bioplastics, due to its unique viscoelasticity and low water solubility [1,2,3,4]. Gluten is a complex, highly cross-linked and three-dimensional network formed of intermolecular covalent bonds (disulphide bonds, dityrisine cross-links, (iso) peptide) and non-covalent interactions (hydrogen, hydrophobic, ionic) [5,6,7]. The structure and properties of the gluten network are determined by both non-covalent and covalent bonds. However, the most influence is exerted by the number and distribution of disulphide bonds (SS), which are dependent on environmental and genetic factors [5]. To compensate these variabilities of gluten properties (e.g., with specific enzymes or chemical agents) as well as to elucidate structure-function relationships in any kind of material (biomaterial or food products), a precise analysis of the network characteristics, especially the degree of cross-linking, is important. However, the network structure is challenging to analyze due to its complexity and low solubility [8,9]. The degree of cross-linking is often characterized by means of the molecular weight of HMW (high molecular weight) and LMW (low molecular weight) glutenin subunits, GMP (glutenin macropolymer) as well as the amount of free sulfhydryl (SH) groups [2,5,10,11]. However, the spatial arrangement of gluten proteins within a complex matrix (present as e.g., clustered agglomerates or homogeneously distributed network) cannot be elucidated sufficiently with these methods. This would support a better understanding of structure-function relationships and the control of processing properties [4]. An appropriate method to visualize the arrangement of gluten microstructure is the confocal laser scanning microscopy (CLSM). While influencing factors on gluten network formation or modifications in cross-linking, like the effect of enzymes or additives, are investigated in detail on a molecular or macroscopic level [10,12,13,14], the microstructure is little analyzed.

Thus, the aim of this study is to clarify if structural changes caused by chemical or enzymatic agents can be detected qualitatively as well as quantitatively on a microstructural level with CLSM. Furthermore, gluten network attributes and various types of polymer arrangements should be specified in general. For this purpose, specific gluten-influencing factors were tested in a model flour-water-system by reduction (glutathione, GLU), oxidation (ascorbic acid, ASC) and enzymatic effects (glucose oxidase, GOX, transglutaminase, TG), each variation in different concentrations. In this context, microstructural protein network characteristics dependent on the modification of cross-linking (disulphide, (iso) peptide, dityrosyl) were considered. The flour-water-system was chosen due to its complex matrix structure comparable to a wheat dough and due to its formation to a complex, spatial arranged network in a multiphase system. Furthermore, gained knowledge about network classifications can be derived for other gluten polymer materials (e.g., bioplastics containing of gluten blends with glycerol, water, polycaprolactone or polylactic acid [15]). Structural attributes of the gluten network’s microstructure were quantified by means of the method protein network analysis (PNA) established by Bernklau et al. [16]. In order to define network classifications in general, further specific as well as unspecific variations on gluten polymer (reduction (RHL) and increase (IHL) of hydration level; addition of rapeseed oil (ROI), shortening (SHO), potassium bromate (KBrO_3_), bromelain (BRN)) were analyzed and correlations of structural attributes (lacunarity, branching rate, end-point rate, protein width and length) were performed.

## 2. Materials and Methods

### 2.1. Materials

Four different German commercial wheat flours Type 550 were supplied by Rosenmühle (Landshut, Germany). Flour characteristics were analyzed according to methods of the AACC international (AACCi) and of the International Association for Cereal Science and Technology (ICC): the moisture (AACCi 44-01), protein content (AACCi 46-16, N × 5.7), ash (ICC 104/1), and falling number (AACCi 56-81). All specifications and characteristics of the four flours are summarized in Table 1. The use of various flours was intended to detect microstructural changes independent of the raw material. Ascorbic acid was supplied by Carl Roth GmbH + Co. KG (Karlsruhe, Germany), glutathione by VWR International GmbH (Darmstadt, Germany), transglutaminase (≥1000 units/g) and glucose oxidase (≥1100 units/g) by AB Enzymes GmbH (kindly provided by AB Enzymes GmbH, Darmstadt, Germany), potassium bromate (KBrO_3_) by ThermoFisher GmbH (Karlsruhe, Germany), bromelain (≥3 units/mg protein) and d(+)glucose by Sigma-Aldrich Chemie GmbH (Steiheim, Germany), Rhodamine B by Merck KGaA (Darmstadt, Germany), rapeseed oil by Cargill Oil Packers bvba (Izegem Belgien) and shortening (ingredients: palm fat, coconut fat, rapeseed oil, water, emulsifier, NaCl, citric acid, aroma, carotin) by MeisterMarken (CSM Deutschland GmbH, Bingen, Germany).

### 2.2. Preparation of Flour-Water-Systems

Each standard flour-water-system (model dough) was produced with commercial wheat flour (c.f. Table 1) and demineralized water. The required kneading time and recipe for the standard flour-water-system of each flour was estimated by the water absorption, moisture (corrected to 14%) and the targeted resistance of 500 Farinograph units in a Z-kneader (doughLAB; Perten Instruments, Hägersten, Sweden) according to AACCi method 54-70.01. The determined kneading times are listed in Table 1. Flour-water-systems were varied by addition of different agents, in each case in increasing concentrations. The unspecific gluten modifications were caused by addition of rapeseed oil (0.0, 5.0, 10.0, 20.0 and 50.0 g/100 g flour_b_), shortening (0.0, 5.0, 10.0, 15.0, 20.0 and 50.0 g/100 g flour_b_), increase of hydration level (59.18 (standard), 64.87, 69.86, 74.85, 79.84 and 89.82 mL water/100 g flour_a_) and reduction of hydration level (reduced water concentration compared to a standard flour-water-system; 57.83 (standard), 53.84, 49.84, 47.58 and 45.85 mL water/100 g flour_b_). Specific gluten modifications were caused by addition of the chemical agents glutathione (0.0, 7.5, 15.0, 30.0, 45.0, 60.0 and 75.0 mg/100 g flour_b_), ascorbic acid (0.0, 25.0, 50.0, 100.0, 150.0 and 200.0 mg/kg flour_c_) as well as potassium bromate (0.0, 60.0, 120.0 and 180.0 mg/kg flour_d_). Enzymatic agents were represented by bromelain (0.0, 200.0, 1000.0, 2000.0, 3000.0, 6100.0 mg/kg flour_d_), transglutaminase (0.0, 100.0, 1000.0, 2000.0, 5000.0 and 10000.0 mg/kg flour_d_) and glucose oxidase (0.0, 20.0, 40.0, 60.0, 100.0 and 150.0 mg/kg flour_d_, addition of 0.5 g glucose/100 g flour_d_ to each sample). Each dough variation was produced in triplicate.

### 2.3. Microstructure Analysis by Confocal Laser Scanning Microscopy

Each sample was stained with Rhodamine B (0.01 g/100 mL water) for CLSM measurement in order to visualize protein microstructure. The dye was kneaded combined with the flour-water-system by replacing 5 mL of bulk water with Rhodamine B solution according to the bulk water technique [17]. An eclipse Ti-U inverted microscope with an e-C1 plus confocal system (Nikon GmbH, Düsseldorf, Germany) with a Plan Apo VC 60x/1.40 oil objective and a 534 nm laser (emission 590/50 nm) were used for visualization. Eight different images were taken of each sample with a resolution of 1024 × 1024 pixel and a size of 215 × 215 µm.

### 2.4. Image Processing and Analysis

Protein network characteristics were quantified by protein network analysis (PNA) according to the method of Bernklau, Lucas, Jekle and Becker [16]. For this purpose, AngioTool64 version 0.6a (National Cancer Institute, National Institute of Health, Bethesda, MD, USA) was applied. Each CLSM image was analyzed with the same settings to ensure a reproducible quantification of the network attributes. The parameters for vessels diameter (implies protein diameter) were set to 3 and 5, intensity low and high threshold to 15 and 255, small particles were removed under 35 and the function “fill holes” was deactivated. Calibration was set to 4.76 pixel/µm.

At least five attributes are required for a detailed characterization of the protein network [18]: the structural network attributes branching rate (number of junctions/protein area; describes the network connectivity), end-point rate (number of end-points/protein area; describes the weakness of a network), average protein length (length of a continuous protein particle) and protein width (thickness of protein threads) as well as the morphological attribute lacunarity (attribute for the amount and size of network gaps; describes irregularities of a structure). A detailed description of the attributes can be found in the publication of Bernklau, Lucas, Jekle and Becker [16].

### 2.5. Statistical Analysis

Results were evaluated statistically with GraphPad Prism 6 (version 6.01, GraphPad Software Inc., La Jolla, CA, USA) with one-way analysis of variance (ANOVA) followed by a Tukey-test. All values are represented with the standard error of the mean (SEM). Multivariate statistics and principal component analysis (PCA) were performed with JMP Pro software (version 12.2.0, SAS Institute Inc., Cary, NC, USA).

## 3. Results and Discussion

Specific as well as unspecific gluten-modifying chemical and enzymatic agents were studied on their effect on the gluten network’s microstructure of flour-water-systems, respectively, in order to define network classifications in general. In the following sections, four representative examples of specific gluten-modifying agents are shown in detail, followed by a multivariate statistic of all 10 variations. The gluten polymer investigations were performed by means of a flour-water-system due to its complex matrix comparable to a wheat dough. For that reason, the discussion is based on studies of wheat products.

### 3.1. Protein Network Formation Modified by Glutathione

**Figure 1:**
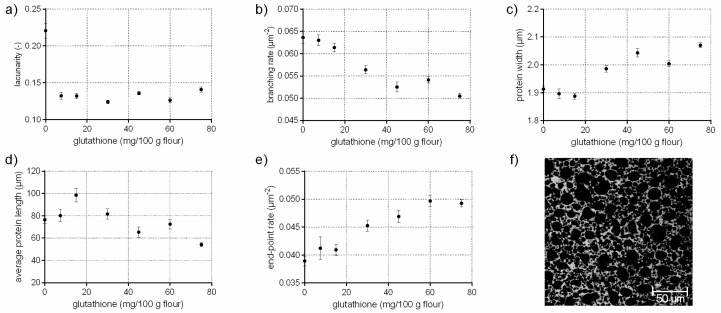
Influence of glutathione on the protein network attributes. Flour-water-systems with increasing concentrations of glutathione were analyzed by confocal laser scanning microscopy (CLSM) followed by protein network analysis (PNA); (**a**) lacunarity; (**b**) branching rate; (**c**) protein width; (**d**) average protein length; (**e**) end-point rate and (**f**) CLSM image (scale 215 × 215 µm, GSH 60 mg/100 g flour). Means are shown with standard error (*n* = 24).

The tripeptide glutathione (γ-glutamylcysteinglycine) promote, as a reducing agent, the interchange of SH with SS bonds. Thus, glutathione causes a cleavage of existing disulphide bonds among proteins resulting in a weakening effect of dough structure [19,20]. This effect has been studied comprehensively on a molecular (disulphide bonds, sulfhydryl interchange) and macroscopic (dough rheology, bread volume and texture) level in literature [20,21,22]. In Figure 1, it is shown that the addition of glutathione had also distinct effect on the microstructure of flour-water-systems. Small gaps are visible in the CLSM micrograph within the protein network due to ruptured protein threads caused by cleaved disulphide bonds (Figure 1f). These findings were also quantified by PNA; the branching rate decreased by 21% at the highest GSH concentration whereas the end-point rate increased significantly by 27% due to a higher rate of open-ended protein threads. The rupture of the disulphide bonds resulted in protein fragments with shorter and thicker protein threads (c.f. Figure 1c,d). The lacunarity decreased by 36% due to the loss of an intact network, which caused closely packed but unconnected protein fragments. These structural changes explain the distinct decrease in dough firmness, which is reported in other studies [20,23].

### 3.2. Protein Network Formation Modified by Ascorbic Acid

**Figure 2:**
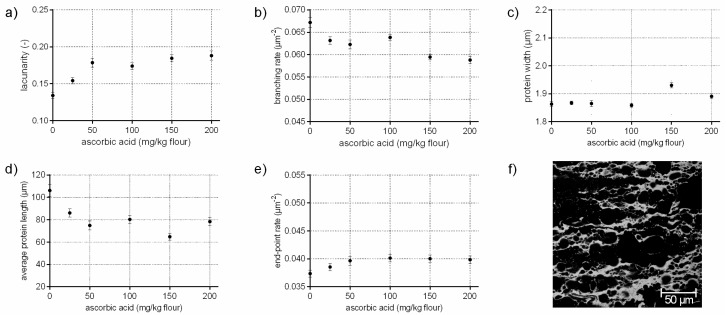
Influence on ascorbic acid on the protein network attributes. Flour-water-systems with increasing concentrations of ascorbic acid were analyzed by CLSM followed by PNA; (**a**) lacunarity; (**b**) branching rate; (**c**) protein width; (**d**) average protein length; (**e**) end-point rate and (**f**) CLSM image (scale 215 × 215 µm, ASC 150 mg/kg flour). Means are shown with standard error (*n* = 24).

Ascorbic acid rapidly oxidizes by atmospheric oxygen or enzymatically to dehydroascorbic acid, which promote the formation of disulphide bonds among gluten proteins by minimizing the exchange of SH/SS of endogenous GSH with intermolecular SS bonds of gluten [12]. Thus, ascorbic acid leads to stiffer and strengthened doughs [23]. On a microscopic level, ASC influences gluten arrangement, differently as expected, by a decreased branching rate of 12%. The average protein length decreased by 26% with increasing ASC concentrations, whereas the end-point rate increased only by 7%. Usually, higher values for the end-point rate indicate a cleavage of bonds and a weakened structure, as it was shown above for GSH. However, the increase of the end-point rate compared to the standard was much higher for GSH flour-water-systems than for ASC samples. These effects indicate, in addition to an increased protein width and higher values for lacunarity (up to 40%), various locally higher aggregations of gluten proteins with increasing ASC concentrations. Taking the visual evaluation of CLSM micrographs into account (Figure 2f), it seems that ASC causes a contraction and inhomogeneous distribution of the proteins, leading to starch accumulations (black areas in Figure 2f). This would explain the high values for lacunarity, the increased protein widths and the locally higher aggregation of proteins. Thus, the contracted and thicker proteins might characterize a highly strengthened network. The influence of ASC on the protein formation is discussed contradictory in literature focused on gluten polymers in wheat dough. A strengthened effect of ASC on dough rheology is reported and an increase of cross-links is expected [12,23]. In contrast, an increase of free thiol groups was determined in the glutenins in other studies [11,24], indicating a decrease of disulphide bonds. In addition, Hanft and Koehler [25] found a decrease in dityrosine cross-links with ASC addition. Even if the degree of dityrosine in wheat dough is quite low, it might have an effect on the gluten polymer. Both studies indicated a decrease in gluten cross-links on a molecular level, which could confirm the decrease in the branching rate of gluten network’s microstructure in the present study. Thus, ASC might not influence gluten cross-linking (or at least not on a microscopic level), but affects gluten arrangement and causes strengthened protein threads.

### 3.3. Protein Network Formation Modified by Glucose Oxidase

**Figure 3:**
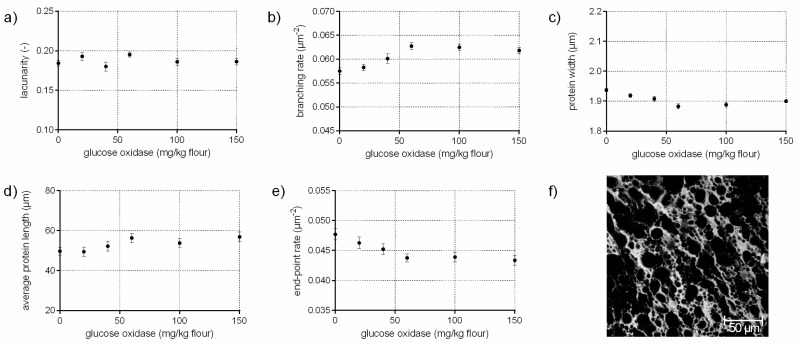
Influence of glucose oxidase on the protein network attributes. Flour-water-systems with 0.5 g glucose/100 g flour and increasing concentrations of glucose oxidase were analyzed by CLSM followed by PNA; (**a**) lacunarity; (**b**) branching rate; (**c**) protein width; (**d**) average protein length; (**e**) end-point rate and (**f**) CLSM image (scale 215 × 215 µm, GOX 100 mg/kg flour). Means are shown with standard error (*n* = 24).

The enzyme glucose oxidase (EC 1.1.3.4) catalyzes, in the presence of oxygen, the oxidation of d-glucose to d-gluconolactone/d-gluconic acid. The by-product hydrogen peroxide (H_2_O_2_) is responsible for the increase in gluten cross-linking by oxidizing thiol groups to disulphide bonds [11,26]. In addition, H_2_O_2_ causes in combination with endogenous wheat peroxidase dityrosine cross-links in gluten significantly [11]. Thus, an increase of the branching rate of gluten on a microstructural level was assumed in this study, which was confirmed quantitatively by PNA (Figure 3b). Furthermore, the significant decrease in end-point rate (9%), higher average protein lengths (14%) as well as the constant low value for lacunarity indicate the presence of a continuously interconnected gluten network with increasing GOX concentrations (c.f. Figure 3d,e). Likewise, the visual evaluation of the CLSM micrograph revealed a homogeneous, highly branched gluten network (Figure 3f). This is in accordance with Steffolani, Ribotta, Pérez and León [10], who determined a decrease of thiol groups, an increase of the glutenin macropolymer and large protein aggregates. Niu et al. [27] reported also a strengthened and stable gluten network with GOX addition due to an increase of β–sheet content and α–helix proportion, measured by FT-IR.

Compared to the addition of ascorbic acid (Section 3.2), which has also an oxidative effect, the gluten network was modified to a completely different extent due to other mechanisms of disulphide cross-linking and dityrosine formation. These results show, that different degree and type of cross-linking cause diverse gluten polymer arrangement.

### 3.4. Protein Network Formation Modified by Transglutaminase

**Figure 4:**
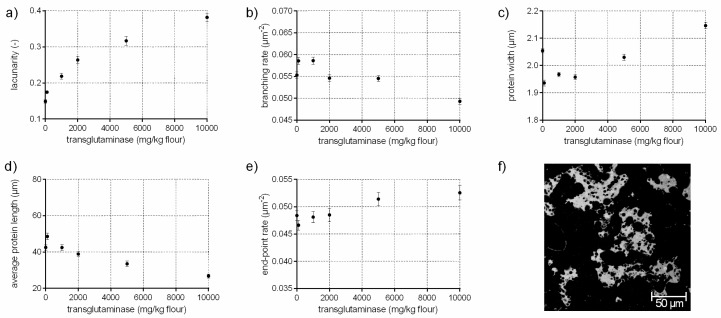
Influence of transglutaminase on the protein network attributes. Flour-water-systems with increasing concentrations of transglutaminase were analyzed by CLSM followed by PNA; (**a**) lacunarity; (**b**) branching rate; (**c**) protein width; (**d**) average protein length; (**e**) end-point rate and (**f**) CLSM image (scale 215 × 215 µm, TG 5000 mg/kg flour). Means are shown with standard error (*n* = 24).

The enzyme transglutaminase (EC 2.3.2.13) catalyzes the cross-linking between lysine and glutamine residues of gluten (the impact on high molecular weight, HMW, is most pronounced). Thus, the development of isopeptide bonds improves dough strength and resistance to extension [28]. This modification in the gluten network can be identified by an increased branching rate of 6% when transglutaminase is added up to 1000 mg/kg flour, quantified with PNA of the CLSM micrographs (Figure 4b). Furthermore, the flour-water-system with TG is characterized of a larger, interconnected gluten network (higher average protein length, lower end-point rate) with thinner protein threads compared to the standard and similar to GOX samples (c.f. Section 3.3). However, the effect of TG varies depending on the concentration, resulting in less elastic doughs at higher TG concentrations [10,29]. The alteration cannot only be observed in rheology, but also in the microstructure. The structure of gluten network changed from a homogeneous, highly branched one to a clustered, agglomerate formation of protein fragments at TG concentrations over 2000 mg/kg flour, visible in Figure 4f. These alterations were also detected quantitatively by PNA. The branching rate decreased by 11% for the highest TG concentration compared to the standard, whereas the end-point rate increased significantly by 9%. Moreover, the agglomerate formation was quantitatively expressed by thicker protein threads (5%) and an enormously decreased average protein length (by 37%). The lacunarity was 2.5 times higher compared to the standard due to larger gaps between the individual agglomerate fragments. An uneven distribution of the proteins at high TG concentrations were also detected visually in other microscopic studies [28]. The agglomerate arrangement of the proteins at high TG levels explain also the findings on a molecular level that the GMP particle size increased, whereas GMP content did not increase [30]. This indicates a rearrangement of the proteins to a clustered agglomerate formation at high TG concentrations caused by new isopeptide bonds. This alterations in the gluten arrangement resulted in rigid and less extensible doughs [28].

Overall, a precise and quantitatively determination of the gluten formation under enzymatic or chemical influences has not been published on a microscopic level before. With the research of this study, not only a quantitative description of different kinds of gluten arrangements could be established; but also microstructural alterations dependent on the modification of cross-linking (disulphide, (iso) peptide, dityrosyl) were elucidated.

### 3.5. Classification of Gluten Polymer Networks

Based on the results of the sections above, it became apparent that various modifications and types of cross-links cause different arrangements of the proteins. However, the interpretation of the five quantitative attributes of PNA, especially the meaning of changes in these attributes in regard of the spatial arrangement of the whole network, is challenging. In order to specify general assertions about structural dependencies and to define different polymer arrangements, correlations of the network attributes were performed.

#### 3.5.1. Effect of Specific Gluten-Modifying Agents

In addition to the four specific gluten-modifying agents of Section 3.1, Section 3.2, Section 3.3 and Section 3.4, further specific agents, bromelain and potassium bromate, were studied on their effect on gluten microstructure. Bromelain, at low concentrations (<1000 mg/kg flour), showed similar effects as glutathione (c.f. supplementary data 1). At higher concentrations, the protein network was destroyed entirely due to the proteolytic activity of bromelain. Thus, the proteins formed a liquid, homogenous mass with embedded starch granules instead of a polymeric network system. This impact caused misleading quantitatively results of the microscopic images. Hence, the results of bromelain concentrations higher than 1000 mg/kg flour were not included for further studies (correlation matrix, PCA). The influence of potassium bromate on gluten polymer was similar to ascorbic acid. Even if most of the activity of potassium bromate takes place during fermentation and baking [31], results showed, that dough microstructure was slightly affected by potassium bromate after kneading. However, the effect of potassium bromate was not as distinct as with ascorbic acid. Results can be found in the supplementary data 2.

**Figure 5:**
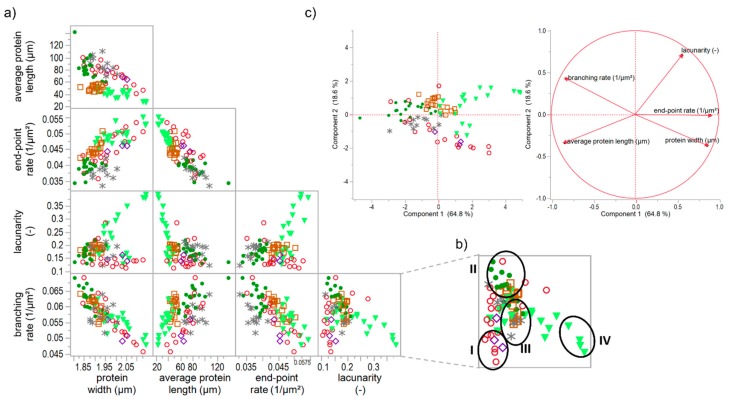
Correlation matrix and principal component analysis of specific gluten modifications. (**a**) Correlation matrix of microstructural attributes of solely gluten-modified flour-water-systems; (**b**) Identification of network types I–IV in the correlation graph of lacunarity vs. branching rate, as an example; (**c**) PCA score and loading plot for the first and second principal component. Symbols: -●- ASC, -∗- KBrO_3_, -◊- BRN, -◯- GSH, -□- GOX, -▼- TG.

When correlating each PNA attribute of all specifically gluten-modified samples, some distinct relation were identified. As visible in Figure 5a, strong linear correlation can be observed for e.g., end-point rate and average protein length (Pearson correlation coefficient r = −0.82) or branching rate and protein width (r = −0.84). Furthermore, the end-pointe rate shows linear correlations with the protein width (r = 0.69) and with the branching rate (r = 0.65). This indicates that the more open-ended protein threads occur, the lower the length of an interconnected protein aggregate, the thicker the proteins threads and the lower the branching rate (up to a weak network structure with contracted protein threads). Inversely, the higher the branching rate, the thinner the protein threads and the larger the interconnected protein aggregate (up to a fully developed network with stretched proteins). These structural relations became also apparent by means of the PCA loading plot in Figure 5c, whereby the correlation of the branching rate and the protein width was most pronounced. While the first component is mainly defined by the structural network attributes (almost 65%), the second component (almost 19%) is dominated by the morphological attribute lacunarity. For this attribute, no linear correlations with the structural network attributes were found. Even if PCA is based on linear correlations, it gives distinct indications that a network classification, in general, is highly influenced by the attribute lacunarity (second component). Hence, the morphological attribute lacunarity separates further network properties. For example, if the branching rate is very low, the lacunarity can be either characterized by very low (0–0.16) or by very high (>0.25) values. The correlation matrix (Figure 5a) of lacunarity vs. branching rate, protein width or end-point rate revealed about four main network types (an example of the network type identification is visualized in Figure 5b):

- A network with a low lacunarity (0–0.16), a very low branching rate, very high end-point rate as well as a high protein width, such as for flour-water-systems with glutathione (c.f. Section 3.1) or bromelain addition (c.f. supplementary data 1).
- A network with a median lacunarity (0.17–0.26), very high branching rate, low end-point rate and a low protein width, such as for a standard flour-water-systems or with glucose oxidase addition (c.f. Section 3.3).
- A network with a median lacunarity (0.17–0.26), low/median branching rate, high end-point rate as well as a high protein width, such as for flour-water-systems with high ascorbic acid (c.f. Section 3.2) or potassium bromate (c.f. supplementary data 2) concentrations.
- A network with a high lacunarity (>0.27), low branching rate, very high end-point rate as well as a high protein width, such as for flour-water-systems with high transglutaminase concentration (<1000 mg transglutaminase/kg four; c.f. Section 3.4).

The terms low or high in the description of the network types are related to each standard flour-water-system. As an interpretation, network type I can be defined as a weak network with only few cross-links, such as disulphide bonds, and as a soft material with a low viscoelasticity. In contrast, network type II describes a highly cross-linked (disulphide and dityrosine bonds), strengthened and homogenously distributed polymer network, perhaps leading to a pronounced viscoelastic material. Network type III is characterized by thicker protein threads, which can be an indication for a strengthened network system even though the degree of cross-links is decreased. However, this only functions in terms of a low or median lacunarity. Compared to network type III, network type IV has a much higher value for lacunarity, a very high end-point rate and protein width. These attributes indicate a clustered agglomerate formation of the protein polymers. Thus, network type IV is more like a particular system than a network. Material properties of a network type IV might be of a high firmness and low viscoelasticity.

#### 3.5.2. Effect of Unspecific and Specific Gluten-Modifying Agents

**Figure 6:**
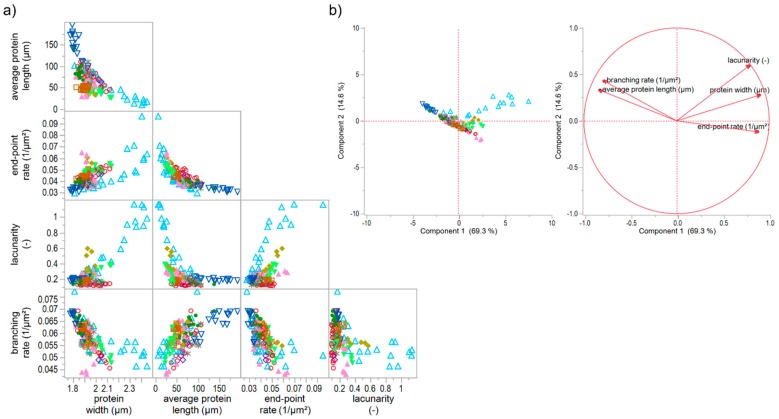
Correlation matrix and principal component analysis of unspecific and specific gluten modifications. (**a**) Correlation matrix of microstructural attributes of all flour-water-system variations; (**b**) PCA score and loading plot for the first and second principal component. Symbols: -●- ASC, -∗- KBrO_3_, -◊- BRN, -▲- SHO, -◯- GSH, -□- GOX, -♦- ROI, -△- IHL, -▽- RHL, -▼- TG.

The effect of unspecific gluten-modifying agents were investigated in combination with the specific ones, in order to extend the assertions about the four network types above. For this purpose, the increase and decrease of hydration level as well as the addition of rapeseed oil and shortening were studied combined with the specific gluten-modifying agents. The increase of hydration level (IHL) caused a weakened gluten network structure due to a dilution effect of water. Detailed results can be found in the manuscript of Bernklau, Lucas, Jekle and Becker [16]. The addition of rapeseed oil (supplementary data 3-ROI) and shortening (supplementary data 4-SHO) caused a similar weakened effect and a formation of clustered protein particles at higher concentrations. However, the lacunarity was much higher for IHL samples and highest concentration of ROI than for SHO ones. This can be explained by a more scattered arrangement of the agglomerates caused by a dilution effect and higher mobility of the particles in IHL and ROI samples. In that case, proteins cannot be stretched during kneading, and a development of a network formation is inhibited, leading to clustered protein particles. This pronounced form of a modified structure is also clearly evident in the correlation matrix and PCA score plot of PNA attributes of all specifically and unspecifically gluten modified samples (Figure 6). The reduction (detailed results visualized in supplementary data 5-RHL) and increase of hydration level mainly caused a shift of a linear correlation for solely specifically gluten-modified samples to an exponential relation of several PNA attributes for specifically plus unspecifically gluten-modified samples. The correlation of e.g., average protein length with the end-point rate (R² = 0.77) or with lacunarity (R² = 0.75) resulted in a non-linear, exponential decay. However, there were also some unclear relations between the attributes or indications for linear relations, but with inconsistencies of IHL values (e.g., protein width vs. branching rate). The PCA score and loading plots revealed that almost 84% of the data could be expressed by the first two components (Figure 6b). As visible in the score plot, most of the IHL data as well as the highest concentrations of ROI and TG were divergent from all remaining data. These data were mainly influenced by increasing lacunarity values, which can be explained by an agglomerate formation or clustered protein particles as described above. This influence was even more distinct for increasing concentrations of IHL samples. Due to the strong dilution effect, the flour-water-systems had a very low viscosity and almost no viscoelastic behavior [16]. Consequently, based on these results, a further network type, V, could be identified, which is an intensified form of network type IV (c.f. Section 3.5.1). Both types, IV and V, describe a particular network formed of protein agglomerates. The difference of both types is, that the protein particles are scattered densely in network type IV and loose in type V, which is confirmed by much higher values for lacunarity. Moreover, the agglomerates of type V are formed due to a plasticizing and dilution effect of the unspecific gluten-modifying agents resulting in unstreched, clustered protein particles, whereas the agglomerate formation of type IV is caused by isopeptide bonds. Based on literature findings for each example (TG for type IV; IHL for type V), network type IV might has a rigid structure, whereas type V is expressed by a very low viscosity.

## 4. Conclusions

In the present study, it was not only clarified that modifications in cross-links (disulphide, (iso) peptide, dityrosyl) of gluten polymer network by enzymatic or chemical agents can be detected precisely and quantitatively on a microstructural level, which has not been published before. It was also shown that findings on a molecular level can be elucidated by microstructural alterations, and that various polymer arrangements can be determined quantitatively by protein network analysis.

Furthermore, a classification of five different types of gluten polymer networks were established on a microstructural level. When solely the gluten network is modified in a complex system (here: flour-water-system) by specific gluten-modifying agents, like protein related enzymes, the structural network attributes branching rate, end-point rate, protein width and average protein length correlated linearly among each other. This revealed the general assertion: the higher the branching rate, the thinner the protein threads and the larger the interconnected protein aggregate. Furthermore, the morphological attribute lacunarity separates the attributes in four main network types: (I) a weak network structure with cleaved and only a few cross-links; (II) a strengthened, highly cross-linked and homogeneously distributed polymer network; (III) a strengthened, less cross-linked network, but with thick protein threads; (IV) a rigid, particular network with clustered agglomerates. These assertions were extended by investigating unspecific gluten-modifying agents, additionally to the specific ones. Thus, a fifth network type was defined: (V) a low viscous, particular network with widely scattered agglomerates. The classification of gluten networks enables a detailed characterization and interpretation of material properties. Gained knowledge about the classifications in a flour-water-system can be derived for other gluten polymer materials, like films, gels, foams or bioplastics. However, individual effects on materials’ mechanical properties should be characterized in the future. Thus, combined with rheological or textural studies, a precise definition of structure-function relationships for gluten polymer materials could be developed.

## Figures and Tables

**Table 1:** Characteristics of the used wheat flours and flour-water-systems. Variations of flour-water-systems were increased hydration level (IHL), addition of rapeseed oil (ROI), shortening (SHO), glutathione (GLU), reduced hydration level (RHL), ascorbic acid (ASC), transglutaminase (TG), glucose oxidase (GOX), potassium bromate (KBrO_3_) and bromelain (BRN).

	**Flour_a_**	**Flour_b_**				**Flour_c_**	**Flour_d_**			
**Ascorbic acid** * (3 mg/ kg flour)	yes	yes				no	no			
**Protein** (g/100 g dry flour)	12.70 ± 0.04	11.49 ± 0.04				11.85 ± 0.04	12.50 ± 0.02			
**Ash** (g/100 g dry flour)	0.63 ± 0.01	0.65 ± 0.01				0.58 ± 0.00	0.64 ± 0.00			
**Falling number** (s)	407.0 ± 14.4	437.3 ± 8.1				434.3 ± 16.8	488.5 ± 18.9			
**Kneading time** ** (s) to 500 FU	180	180				300	240			
	**Flour-Water-Systems**									
	**Flour_a_**	**Flour_b_**				**Flour_c_**	**Flour_d_**			
**Variations**	**IHL**	**ROI**	**SHO**	**GLU**	**RHL**	**ASC**	**TG**	**GOX**	**KBrO_3_**	**BRN**
**Moisture** ** (g/100 g flour)	14.17 ± 0.03	13.92 ± 0.01	13.92 ± 0.01	13.91 ± 0.02	14.13 ± 0.3	14.86 ± 0.07	14.28 ± 0.15	14.14 ± 0.08	13.86 ± 0.04	13.86 ± 0.04
**Water addition** (mL/100 g flour)	59.18	58.32	58.32	57.76	57.83	56.17	60.49	61.06	61.36	61.36
**Kneading time** *** (s) to 500 FU	180	180	180	180	180	300	250	250	240	240

* Commercial wheat flour usually contains ascorbic acid, ** moisture content of flour varies during storage and was determined for each sample variation, *** rounded to the nearest tens.

## Supplementary Materials

The following are available online at http://www.mdpi.com/2073-4360/10/6/617/s1, Figure S1: Influence of bromelain on the protein network attributes, Figure S2: Influence of potassium bromate on the protein network attributes, Figure S3: Influence of rapeseed oil on the protein network attributes, Figure S4: Influence of shortening on the protein network attributes, Figure S5: Influence of a reduced hydration level on the protein network attributes.
