# Supplementary material for: Gluten Polymer Networks—A Microstructural Classification in Complex Systems
Source: Polymers (Basel). 2018 Jun 5;10(6):617. doi: 10.3390/polym10060617 (PMC6403851; doi:10.3390/polym10060617)
Supplement: Supplementary file 1 [file polymers-10-00617-s001.pdf]

Supplementary data

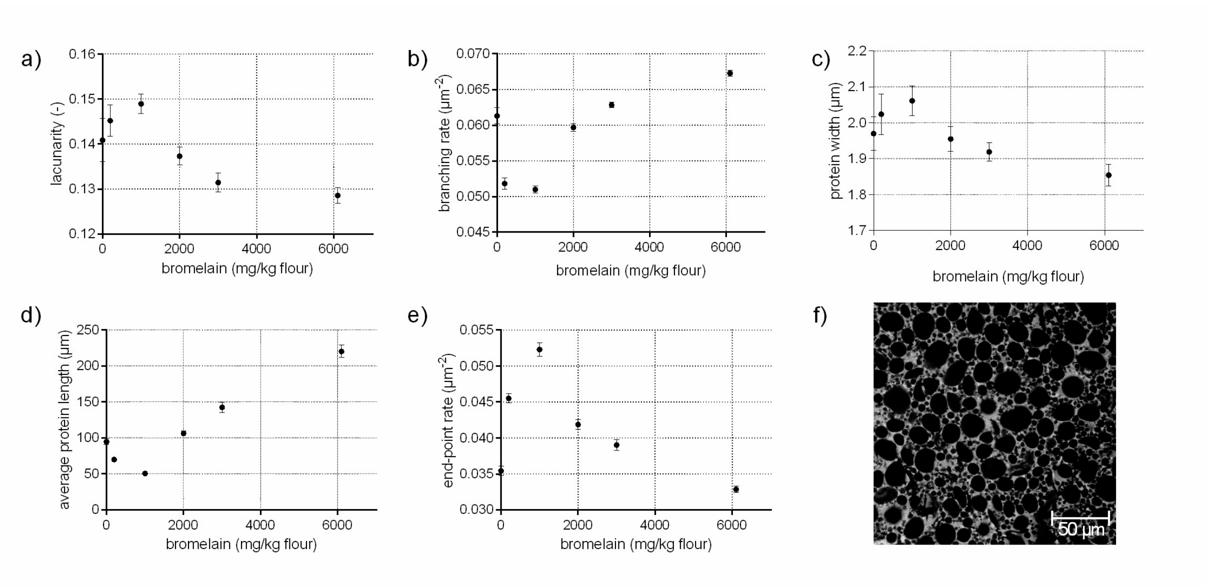

Figure S1. Influence of bromelain on the protein network attributes. Flour-water-systems with increasing concentrations of bromelain were analysed by CLSM followed by PNA; a) lacunarity, b) branching rate, c) protein width, d) average protein length, e) end-point rate and f) CLSM image of an entirely destroyed protein polymer structure by proteolysis (scale 215x215 µm, BRN 6100 mg/kg flour). Means are shown with standard error (n=24).

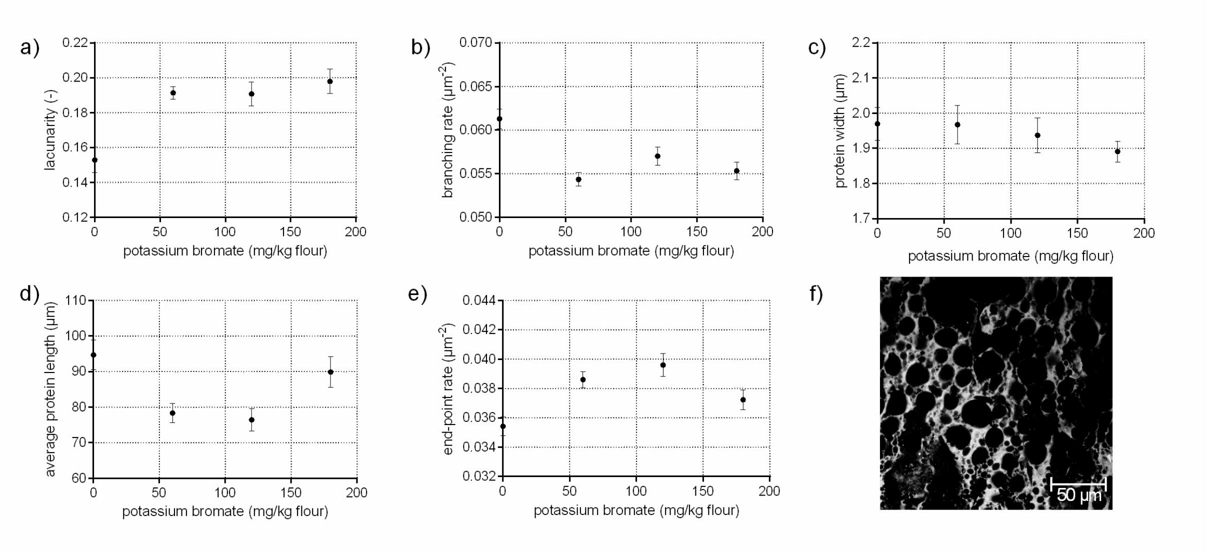

Figure S2. Influence of potassium bromate on the protein network attributes. Flour-water-systems with increasing concentrations of potassium bromate were analysed by CLSM followed by PNA; a) lacunarity, b) branching rate, c) protein width, d) average protein length, e) end-point rate and f) CLSM image (scale 215x215 µm, KBrO3 120 mg/kg flour). Means are shown with standard error (n=24).

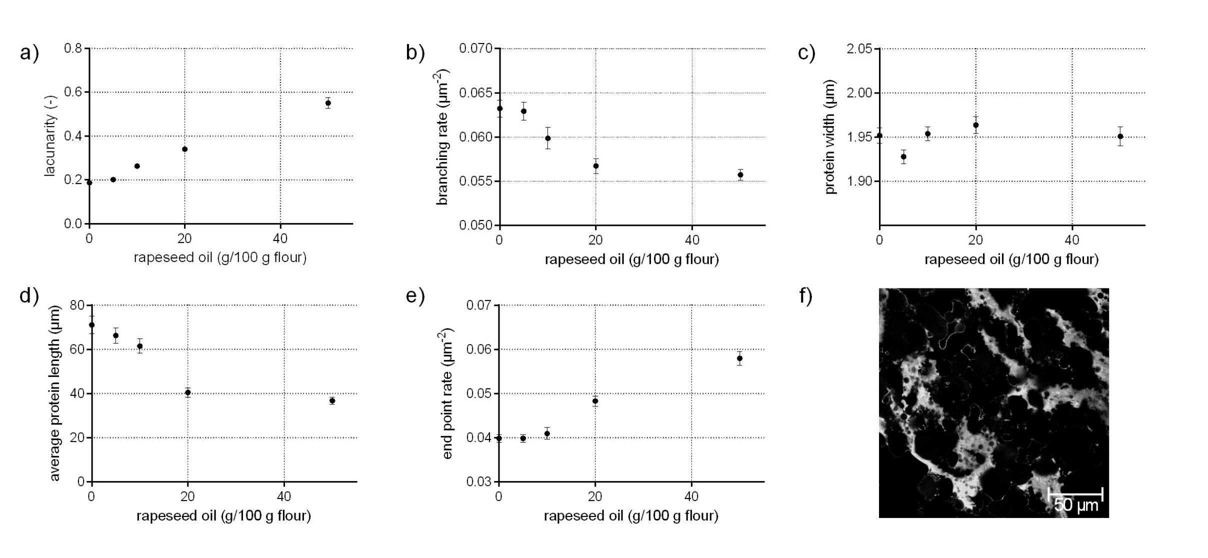

Figure S3. Influence of rapeseed oil on the protein network attributes. Flour-water-systems with increasing concentrations of rapeseed oil were analysed by CLSM followed by PNA; a) lacunarity, b) branching rate, c) protein width, d) average protein length, e) end-point rate and f) CLSM image (scale 215x215 µm, ROI 50 g/100 g flour). Means are shown with standard error (n=24).

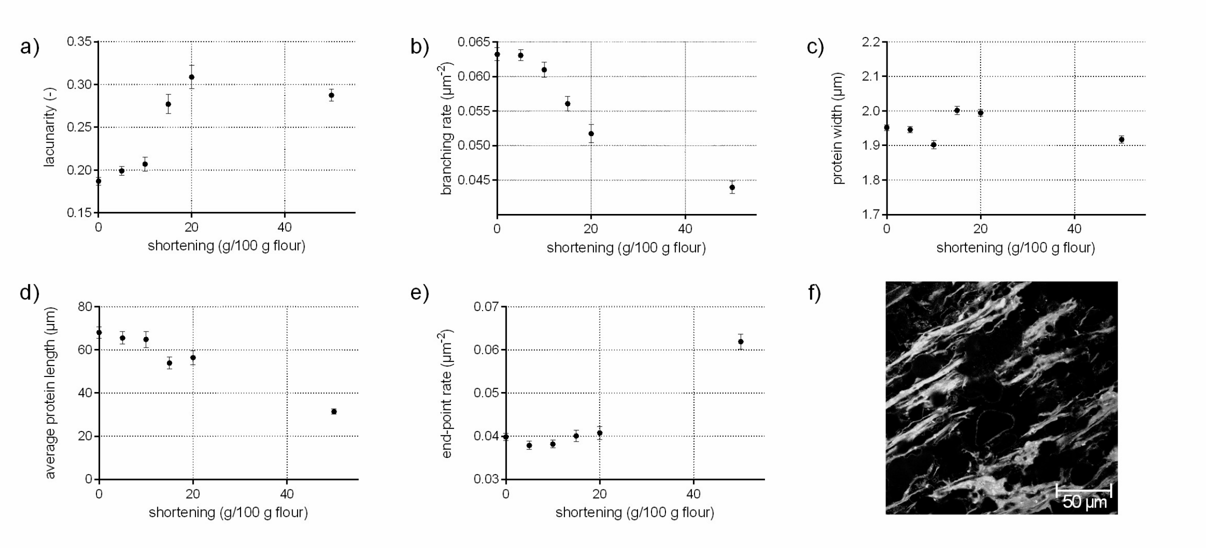

Figure S4. Influence of shortening on the protein network attributes. Flour-water-systems with increasing concentrations of shortening were analysed by CLSM followed by PNA; a) lacunarity, b) branching rate, c) protein width, d) average protein length, e) end-point rate and f) CLSM image (scale 215x215 µm, SHO 50 g/100 g flour). Means are shown with standard error (n=24).

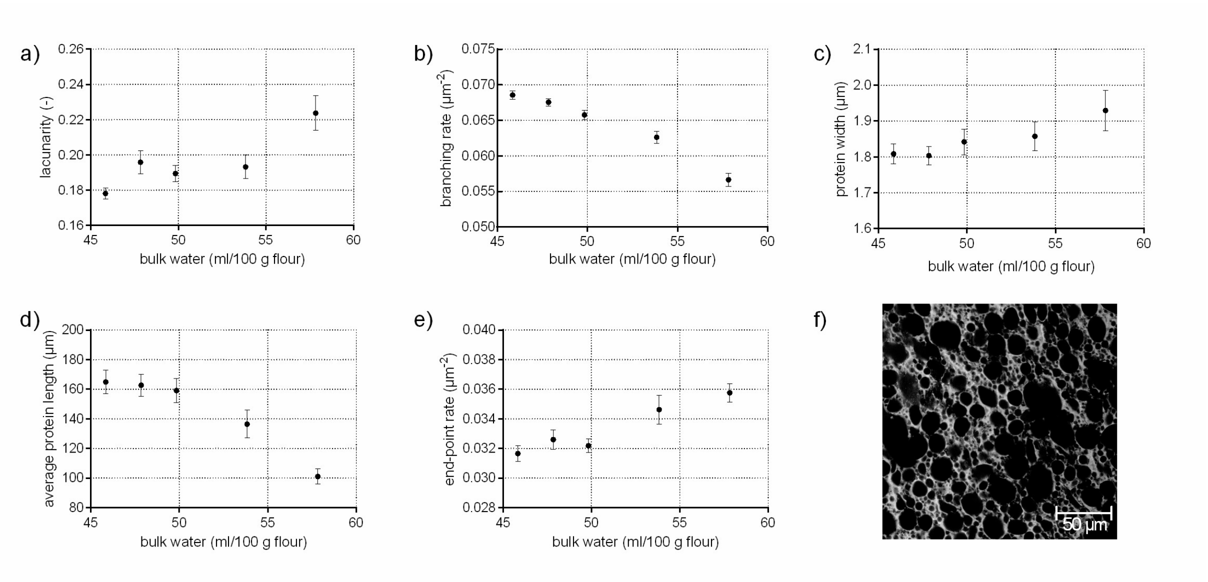

Figure S5. Influence of a reduced hydration level on the protein network attributes. Flour-water-systems with decreasing water hydration levels were analysed by CLSM followed by PNA; a) lacunarity, b) branching rate, c) protein width, d) average protein length, e) end-point rate and f) CLSM image (scale 215x215 µm, RHL 45.85 ml/100 g flour). Means are shown with standard error (n=24).
